# Metabolomics Highlights Reduced Glutamate and Acetylcarnitine in the Amniotic Fluid of Cytomegalovirus-Infected Pregnant Women

**DOI:** 10.1021/acs.jproteome.5c01108

**Published:** 2026-03-17

**Authors:** Michele Costanzo, Marco Miceli, Ilaria Campesi, Sabrina Bianco, Laura Letizia Mazzarelli, Sonia Migliorini, Laura Sarno, Rita Malesci, Serena Salomè, Francesco Raimondi, Maurizio Guida, Giuseppe Maria Maruotti

**Affiliations:** † Department of Molecular Medicine and Medical Biotechnology, 9307 University of Naples Federico II, 80131 Naples, Italy; ‡ CEINGE-Biotecnologie Avanzate Franco Salvatore, 80145 Naples, Italy; § Department of Neuroscience, Reproductive Sciences and Dentistry, University of Naples Federico II, 80131 Naples, Italy; ∥ Laboratory of Sex-Gender Medicine, National Institute of Biostructures and Biosystems, 07100 Sassari, Italy; ⊥ Department of Biomedical Science, University of Sassari, 07100 Sassari, Italy; # Department of Translational Medical Sciences, Division of Neonatology, University of Naples Federico II, 80131 Naples, Italy; ∇ Department of Public Health, University of Naples Federico II, 80131 Naples, Italy

**Keywords:** cytomegalovirus, metabolomic profiling, amniotic fluid, glutamate metabolism, acetylcarnitine, fetal programming

## Abstract

Cytomegalovirus (CMV) is a major cause of morbidity in immunocompromised individuals and represents the leading infectious cause of neonatal congenital deafness. When acquired during pregnancy, CMV can be vertically transmitted to the fetus, potentially resulting in permanent sequelae characterized by intellectual and neurosensory impairments. To investigate metabolic alterations associated with primary maternal CMV infection, we conducted a metabolomics-based analysis of amniotic fluid (AF) from pregnant women who acquired CMV infection during the first trimester, as confirmed by IgG seroconversion, IgM positivity, and low-to-moderate IgG avidity indices. Our findings revealed that the AF metabolomic profiles from CMV transmitter and nontransmitter mothers were remarkably similar. In contrast, both the CMV-exposed groups showed profound metabolic dysregulation compared to uninfected controls, suggesting that CMV-related metabolic disparities may persist irrespective of vertical transmission. Specifically, we observed a significant downregulation of glutamate (*p* < 0.0001) and acetylcarnitine (*p* < 0.0001) in CMV groups compared to control AF samples. Notably, the combined reduction of these two metabolites emerged as a surrogate biomarker signature of recent primary CMV infection of the mother, indicating that AF from both transmitting and nontransmitting pregnancies may share common metabolic adaptations. These alterations may reflect early perturbations of neurobiochemical pathways with unknown effects on babies negative at birth, supporting the need for risk assessment and clinical monitoring even in the absence of congenital CMV infection.

## Graphical abstract

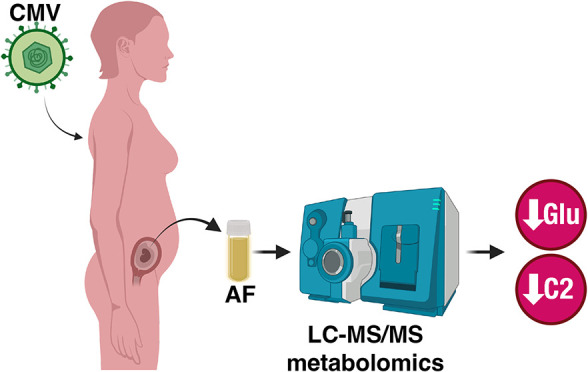

## Introduction

Cytomegalovirus (CMV) is a ubiquitous infectious agent that causes significant morbidity in immunocompromised hosts and is also the most common cause of congenital infection in developing countries within the range of 0.5–2% of all live births.^1–4^ It is estimated that 40 to 80% of the population suffer from CMV infection, which usually evolves without symptoms and results in a latent infection (https://www.epicentro.iss.it/en/cytomegalovirus/). Human CMV (HCMV) is transmitted through direct person-to-person contact via oropharyngeal secretions, urine, semen, milk, tears, blood, and cervical and vaginal secretions. Due to its strict species specificity, humans represent the only natural reservoir for HCMV. Although the earliest events of HCMV transmission remain largely unknown, vertical transmission from mother to fetus occurs via placental circulation, where the complex interactions between the virus, the uterine microvasculature, maternal decidual leukocytes, and invasive interstitial fetal cytotrophoblasts within the maternal decidua may ultimately determine the outcome of infection.^1,5^ The placenta is the first fetal organ to be infected, initially acting as a barrier to the virus, aided in its action by natural maternal immunity.^6^ Then, the virus may infect the fetus and be excreted into the amniotic fluid (AF) through fetal urine once the fetal renal system becomes functional (between the 5th and 12th week).^3^ However, the mechanisms underlying vertical transmission or the existence of protective factors are still being actively investigated to explain the biological variability observed among affected pregnancies.^7^

CMV is an enveloped, double-stranded DNA virus member of the herpesvirus family. Following primary infection (first contact with the virus), CMV can persist in a latent state within the infected cells. Reactivation of CMV from latency to the lytic phase is a complex, epigenetically regulated biological process that involves viral DNA replication and progeny production, resulting in viral shedding and potential transmission to susceptible hosts.^1–3,5,8^ In primary maternal infections, transmission to the fetus occurs in 30–40% of cases, while the risk with recurrent infections or reinfections is 0.15–1%.^4,9^ Most studies highlight the critical impact in high-income countries of primary maternal CMV infections on newborn health than nonprimary infections; however, HCMV infection is nearly universal in early childhood in developing countries, and congenital transmission from seropositive mothers remains common and significantly threatens fetal health.^10–13^ The International Society of Ultrasound in Obstetrics and Gynecology recommends delaying amniocentesis for at least 8 weeks after the presumed maternal infection but not before the 20th week of gestation, while waiting for fetal diuresis.^14^ Prenatal diagnosis of CMV infection can modify pregnancy and neonatal management, including *in utero* antiviral treatment with valaciclovir.^15,16^ Free viral DNA levels >10^5^ copies/mL in AF are considered predictive of symptomatic congenital infection,^17^ whereas values around 10^3^ copies/mL are usually associated with asymptomatic cases at birth.^18^ The most reliable maternal prognostic factor of symptomatic events is the gestational age (GA) at infection.^16^ Approximately 90% of congenitally infected fetuses are asymptomatic at birth, while the remaining 10% present with clinical signs such as growth restriction, jaundice, hepatosplenomegaly, microcephaly, intracranial calcifications, chorioretinitis, thrombocytopenia, neurodevelopmental impairment, or, in severe cases, perinatal death.^4,19,20^ In addition, long-term sequelae of congenital HCMV infection include sensorineural hearing loss (SNHL) and intellectual disability.^21,22^ SNHL can range from mild to severe and can be unilateral or bilateral; up to 50% of hearing loss cases are late-onset and therefore will not be detected through newborn hearing screening but can appear even years after birth.^7,23^

The maternal environment and the fetus are structurally and functionally related by barriers such as placenta that provide regulation for a healthy development as well as mechanical defense and active protection from external agents.^24^ Other barrier systems such as the blood–brain barrier (BBB) are vital for the protection and development of the brain and the effective functioning of the central nervous system (CNS) through the regulation of transport mechanisms.^25–27^ Endothelial tight junctions into the BBB develop early around 8–10 weeks of gestation, allowing firm regulation of concentration gradients between blood and brain.^28^ Although studies on CMV infection in the brain during congenital infection are primarily limited to histopathological and observational analyses,^29^ it is assumed that CMV can enter the CNS either in cell-free or cell-associated forms, but the precise mechanism of BBB crossing remains unidentified.^30^

There is scientific consensus that the BBB remains functionally immature until the mid-second trimester or later, with progressive maturation continuing into late gestation and even postnatally,^31–33^ thus the fetal brain may remain relatively permissive during the early second trimester to endogenous and exogenous compounds, including neuroactive metabolites such as glutamate (Glu).^32,34,35^

This supports the possibility that fluctuations in AF metabolite concentrations may mirror, at least in part, variations in the fetal neurochemical milieu. Glu is an excitatory neurotransmitter essential for neuronal excitability, synaptic plasticity, immunity, and cognitive processes.^36,37^ Its availability has been shown to influence fetal neurological development and immune function in vitro.^38^

In this context, maternal CMV infection may exacerbate metabolic dysregulation within critical developmental windows, when BBB immaturity may allow for a tighter interaction between the fetal brain and the surrounding amniotic environment. Consequently, AF composition at the time of amniocentesis might represent the downstream trace of earlier inflammatory or metabolic events.^39^ This view is consistent with the concept of fetal programming, whereby transient prenatal exposures reshape developmental pathways with effects that may become evident only later in life.^40,41^ Altered Glu homeostasis at the maternal–fetal interface may therefore contribute to long-term neurodevelopmental vulnerability, even in the absence of immediate clinical manifestations.^42–45^

In this work, we used metabolomics to analyze the AF of first-trimester pregnant women infected by HCMV. As amino acids (AA) and acylcarnitines (AC) are key metabolites involved in processes such as protein synthesis, energy metabolism, and mitochondrial function, changes in their abundance can reflect alterations in metabolic pathways that are critical during fetal development or potentially modulated by virus infection.^46,47^ Here we show a marked reduction in AF metabolite levels, including Glu and acetylcarnitine. Our data suggest that CMV effects on AF metabolome may persist even after resolution of primary maternal infection or in the absence of documented transmission to the fetus. Although the functional consequences of these changes remain to be fully elucidated, such metabolic remodeling could potentially influence fetal development. Further studies are needed to clarify whether and how these alterations might affect processes, such as synaptic pruning and neuronal maturation.

## Experimental Section

### Study Design and Population

A prospective, single-center, correlational observational research study was conducted between parturients with primary maternal CMV infection in the first trimester of pregnancy compared to uninfected controls (negative TORCH test). This research received institutional approval from the Ethics Committee of the University of Naples Federico II (Committee protocol number: 301/21). All studies were performed following the Declaration of Helsinki.

All AF samples were obtained by invasive amniocentesis performed to diagnose transmission to the fetus, at least 6 weeks after seroconversion of infected mothers, between 20 and 24 weeks. Invasive amniocentesis in the control group (CTRL) was performed between 16 and 18 weeks of gestation due to the advanced maternal age for the assessment of the risk of chromosomal abnormalities in the fetus.

In total, 24 pregnant women were enrolled at the Infectious Diseases in Pregnancy Clinic of the Department of Neurosciences, Reproductive and Odontostomatological Sciences of the AOU Federico II, Naples, from January 2021 to December 2023. Inclusion criteria for CMV-exposed women included primary maternal infection under the following conditions: CMV-specific IgG seroconversion; virus-specific IgM antibodies and low or moderate IgG avidity index (AI); CMV-DNA in maternal blood; no ongoing disease at the time of amniocentesis; signed informed consent; and age over 18 years. The exclusion criteria were: high IgG avidity; patients who refused amniocentesis; patients who did not sign the informed consent; patients with positive TORCH tests for one or more infections in addition to CMV; other diseases detected; and age less than 18 years. For control women inclusion criteria were: advanced age; negative TORCH test for all infections; and no ongoing disease at the time of amniocentesis. Exclusion criteria were: no advanced age; low-risk pregnancy; patients who had refused amniocentesis; patients who had not signed informed consent; patients with positive TORCH tests for one or more infections, other diseases detected; age less than 18 years.

Globally, our cohort included 14 uninfected women (CTRL) and 10 women who contracted primary CMV infection in the first trimester of pregnancy. Of these, two subjects showed positivity to CMV-DNA in AF after amniocentesis and were labeled as “transmitters”, while eight subjects showed negativity to CMV-DNA in AF and were labeled as “nontransmitters”. Finally, as additional samples from the previously examined groups were unavailable, we obtained serum samples from an independent cohort of eight pregnant women in the same gestational periods as explained above and divided as CMV-positive (*n* = 4) and CTRL (*n* = 4).

### Diagnosis of Primary Maternal CMV Infection and Fetal Infection

Diagnosis of primary CMV infection is based on the presence of at least two of the following criteria: CMV-specific IgG seroconversion; virus-specific IgM antibodies and IgG AI; and CMV-DNA in maternal blood. In most pregnant women, the timing of maternal infection was determined by CMV-specific IgG seroconversion (considering a 1–2-month interval between the last seronegative result and the first seropositive result in the serum sample) and/or the presence of specific IgM, low AI, and *de novo* appearance of neutralizing antibodies in human embryonic fibroblasts, together with clinical signs and symptoms. When no signs/symptoms were reported, the kinetics of IgG, IgM, and AI over time were analyzed to identify the onset of maternal infection. CMV transmission was demonstrated by the detection and quantification of viral DNA in AF. The results of prenatal diagnosis were confirmed in newborns at birth by analyzing their urine for the PCR detection of CMV-DNA.

### Sample Preparation and Targeted LC-MS/MS Analysis

A targeted liquid chromatography-tandem mass spectrometry (LC-MS/MS)-based approach was employed to identify and quantify amino acids (AA) and acylcarnitines (AC) in AF and serum specimens.^48,49^ Sample preparation procedures and metabolite extraction from AF are the same as reported.^50^ Briefly, AF samples were centrifuged at 250*g*, 5 min, and 4 °C to remove floating cells. Then, 10 μL of AF supernatants or serum samples were spotted on a filter paper, and metabolites were extracted with 200 μL of methanol containing stable isotope-labeled AA and AC standards. Metabolites and internal standards were derivatized with 80 μL of n-butanol/3 N HCl (30 min, 65 °C) and dried under nitrogen flow. A solution of acetonitrile/water (70/30) with 0.05% formic acid was added prior to injection into the chromatographic system via flow injection analysis (FIA). The LC-MS/MS platform consisted of a 1260 Infinity II HPLC instrument (Agilent Technologies, Waldbronn, Germany) coupled with a 5500+ QTRAP mass spectrometer (SCIEX, Framingham, MA). Compounds were targeted in positive ionization mode by precursor ion scan, neutral loss scan, or multiple reaction monitoring. The analytes were identified and quantified using the Analyst v1.7 and ChemoView v1.2 software (SCIEX) through comparison of analyte areas with those of stable isotope-labeled internal standards and finally expressed as μM. MS runs were acquired three times for each sample to increase the statistical reproducibility of the data and the robustness of the biological findings. The accuracy and precision of our MS platform are evaluated in each set of analyses using quality control (QC) samples, prepared at four different concentrations (low, mid, high, and very high), provided by the Center for Disease Control and Prevention (Atlanta, GA). In addition, blanks were included after triplicate runs and between different conditions. The raw data files (.wiff) acquired by LC-MS/MS were converted to the mzML format by ProteoWizard software for further use.

### Metabolomics Data Analysis

The metabolomic data set was processed with the MetaboAnalyst 6.0, GraphPad Prism 10.0, and SRplot tools for chemometrics and statistical analyses.^51–53^ The technical triplicates of each sample were kept as individual features for most of the analyses reported. The data were imported in MetaboAnalyst and normalized (log_10_-transformed and autoscaled) for multivariate statistics. Principal component analysis (PCA), orthogonal partial-least-squares discriminant analysis (OPLS-DA), and PLS-DA models were generated to detect the level of variance in the analyzed groups. Samples that deviated significantly from the main data cluster upon unsupervised PCA were recognized as outliers and excluded from the data set. The statistical significances of PCA group patterns (*p*-value based on 999 permutations) were evaluated by Permutational Multivariate Analysis of Variance (PERMANOVA) using the Euclidean distance based on the PCs to compute the distance. OPLS-DA model performance was evaluated using the fitness of model (R2Y) and the predictive ability (Q2) values.^54^ PLS-DA analysis was used to predict the important metabolites able to discriminate groups as indicated by the Variable importance in Projection (VIP) score, which is a weighted sum of squares of the PLS loadings.^55^ A threshold of VIP > 1.5 was set to select important VIP metabolites. PLS-DA was performed with the 5-fold cross-validation method using five components for the classification, checking the fitness (R2) and the accuracy of the model as performance measures. Metabolite Set Enrichment Analysis (MSEA) was performed by Over Representation Analysis using the VIP metabolites as input and the RaMP-DB (integrating KEGG via HMDB, Reactome, WikiPathways) as pathway database. Receiver operating characteristic (ROC) curves were generated using the Biomarker Analysis module of MetaboAnalyst 6.0 and the Multivariate ROC curve-based exploratory analysis (Explorer) feature. ROC curves were obtained by MonteCarlo cross-validation (MCCV) with balanced subsampling useful when working with limited sample size.^56^ During each MCCV iteration, two-thirds of the samples were selected for evaluating feature importance. The top-ranked features determined by the PLS-DA algorithm were successively used to create classification models that were validated on the left-out samples. This process was iteratively executed multiple times to determine the performance and confidence intervals of the models. The results of ROC curve analysis are reported as the area under the curve (AUC) generated from the model based on molecule area under ROC curve (AUROC), T-statistics, and log_2_ fold change, and the 95% confidence interval is calculated using 500 bootstrappings. Raw or transformed concentration data were used for univariate statistics. Hierarchical clustering analysis and heatmaps were generated with SRplot using Euclidean distance. Volcano plot analyses were carried out by multiple unpaired parametric *t*-test with Welch correction. A significance threshold based on the False Discovery Rate at 1% (−log10 *q*-value >2) with the two-stage linear step-up procedure of Benjamini, Krieger, and Yekutieli was chosen for binary comparisons. Multiple comparisons were carried out by ordinary one-way ANOVA considering as significant *p*-values <0.05. Binary comparisons of single molecules were carried out by unpaired *t*-test considering as significant *p*-values < 0.05. The AF/serum ratio was calculated for both CMV and CTRL groups as the ratio between the mean metabolite concentrations measured in AF and serum, with values normalized to the mean concentration of the respective control group. Delta (Δ) values were calculated by subtracting the CMV normalized mean metabolite level from that of the CTRL group. Correlation analysis was performed computing Pearson correlation and simple linear regression after testing normality of the distributions with D’Agostino & Pearson test.

## Results

### Profiles of Maternal Parameters and Newborn Outcomes upon CMV Exposure

The age of CMV-exposed patients (*n* = 10) at the time of amniocentesis ranged from 26 to 34 years with a mean of 29.5 years, while healthy controls (CTRL, *n* = 14) who underwent amniocentesis for cytogenetic analysis showed a mean of 37.5 (35–42 years). The mean gestational age (GA) at the first visit for CMV-exposed women was 13.4 weeks, while GA at the time of infection was estimated at 12.6 weeks; for controls, the mean GA at the first visit was 14.1 weeks. GA at amniocentesis was 20.8 and 17.7 weeks for CMV-exposed and CTRL, respectively (Table 1).

**Table 1:** Main Demographic Characteristics of the Enrolled Subjects

	CMV-positive (mean)	CTRL (mean)
Age of pregnant women (years)	29.5 y	37.5 y
Gestational age at first visit (weeks)	13.4 w	14.1 w
Estimated week of pregnancy at infection (weeks)	12.6 w	
Gestational age at the start of antiviral therapy (weeks)	16.2 w	
Gestational age on neurosonography (weeks)	29.6 w	
Gestational age at amniocentesis (weeks)	20.8 w	17.7 w

CMV-exposed women were subdivided according to efficient vertical transmission of CMV as transmitters (*n* = 2) or nontransmitters (*n* = 8). Data relative to clinical, virological, and biochemical parameters for both groups were reported in Table 2. In particular, primary maternal CMV infection was diagnosed by IgG seroconversion in all cases, while the 90% still present positive IgM antibodies. All the cases were characterized by an unknown onset of infection, which was identified by the avidity index kinetics of IgM and IgG antibodies. All patients but one, who refused the therapy by choice, underwent valacyclovir treatment. Transmitters were confirmed by positive amniocentesis, with a high viral load (on average 43 × 10^5^ copies/mL CMV-DNA) in the two fetuses who were symptomatic at birth. Negative AF viral loads were found in nontransmitters cases, whose fetuses were then negative at birth. One newborn of the mother who refused valacyclovir therapy presented neurological sequelae in terms of unilateral SNHL (Table 2).

**Table 2:** Profile of Pregnant Women with CMV Infection and Observed Distribution of Newborn Outcomes^a^

virological, biochemical, and clinical parameters	Transmitters n/N	Nontransmitters n/N	% total cases
First-trimester infection	2/2	8/8	100.0
CMV IgG seroconversion	2/2	8/8	100.0
CMV IgM+	2/2	7/8	90.0
Low IgG avidity	1/2	4/8	50.0
Moderate IgG avidity	1/2	4/8	50.0
Maternal urine positive for CMV-DNA	2/2	6/8	80.0
Maternal blood positive for CMV-DNA	2/2	2/8	40.0
Adherence to amniocentesis	2/2	8/8	100.0
Adherence to valacyclovir	1/2	8/8	90.0
Positive CMV-DNA in amniotic fluid in symptomatic newborns	2/2	0/8	20.0
Negative CMV-DNA in amniotic fluid in asymptomatic newborns	0/2	8/8	80.0
Negative neurosonography	2/2	8/8	100.0
Positive CMV-DNA in newborn’s urine	2/2	0/8	20.0
Negative CMV-DNA in newborn’s urine	0/2	8/8	80.0
Positive infectious disease screening in the newborns	2/2	0/8	20.0
Prevalence of symptomatic CMV infection at birth	2/2	0/8	20.0
Sensorineural deafness in the newborns	1/2	0/8	10.0
Other sequelae	1/2	0/8	10.0

a IgG, immunoglobulinG; IgM, immunoglobulinM; n/N, number of cases/total cases for that condition.

### Amniotic Fluids of CMV-Infected Women Showed Profound Amino Acid Dysregulations

In this work, we profiled the AF metabolome of pregnant women diagnosed at the first trimester with CMV infection. MS-based targeted metabolomics analysis allowed the quantification of 50 analytes, including 12 amino acids (AA) and 38 acylcarnitines (AC). First, we aimed at investigating the AF differences between clear-cut CMV transmission and no evidence of *in utero* transmission. Unsupervised multivariate principal component analysis (PCA) and orthogonal partial-least-squares discriminant analysis (OPLS-DA) models did not reveal differentiation between transmitters and nontransmitters groups. PCA through PERMANOVA statistics showed a low separation of the two subgroups (*F*-value = 3.8755; *p*-value = 0.027) (Figure 1A), while the OPLS-DA revealed this model to have weak fitness (R2Y = 0.557) and low predictive ability (Q2 = 0.325) (Figure 1B). Indeed, volcano plot analysis showed the significant (*q* < 0.01) decrease of only three metabolites, namely Orn, Xle, and C4, in transmitters compared to nontransmitters AF (Figure 1C and Table 3). Hierarchical clustering based on the differentially abundant metabolites did not show a sharp separation of the two groups (Figure 1D). We concluded that the metabolome is not strongly changing between transmitters and nontransmitters, proposing that CMV effects may be reflected in AF even after resolution of primary infection or in the absence of documented transmission to the fetus.

**Figure 1:**
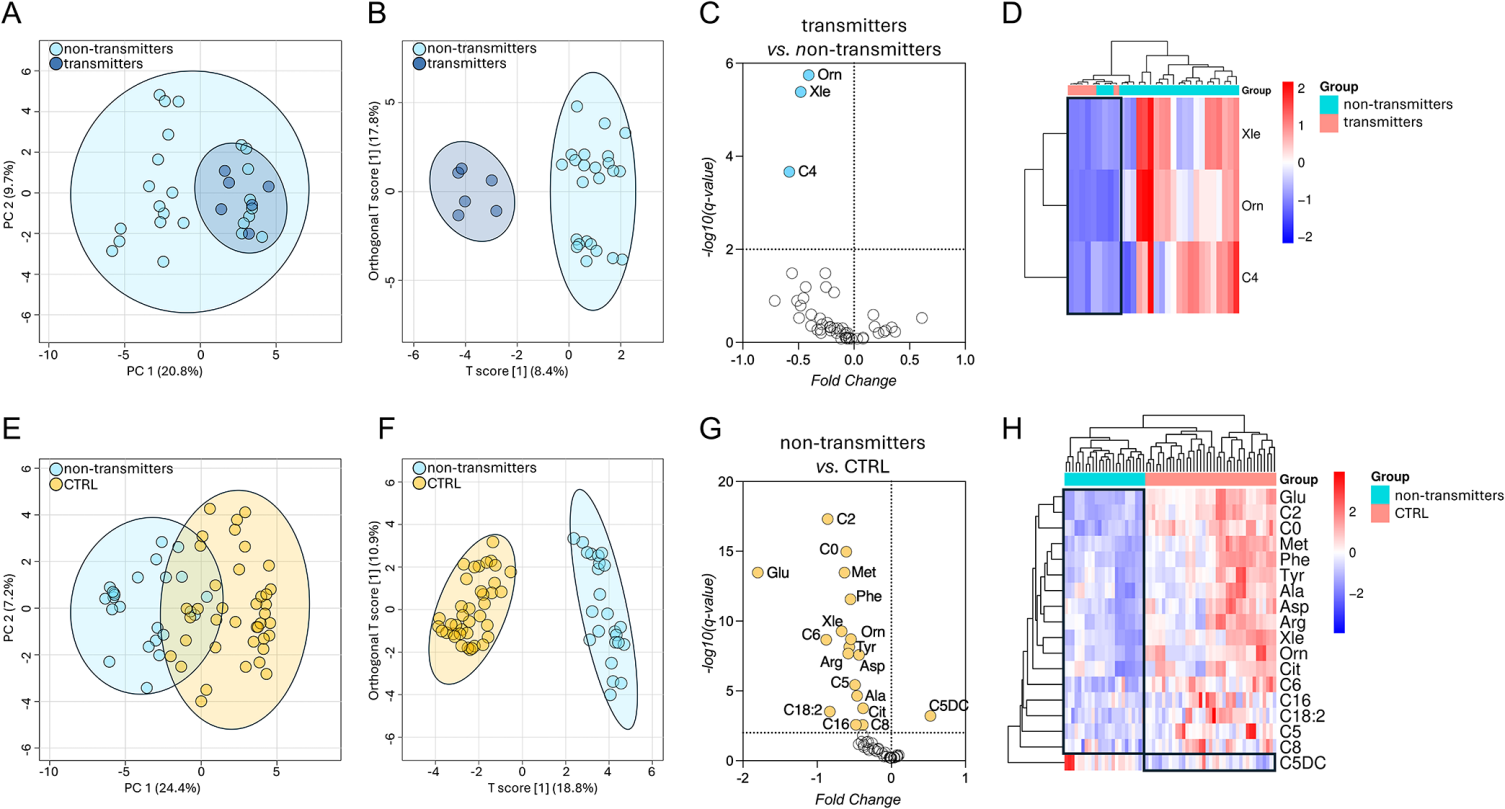
Metabolomics profiling of CMV-infected and noninfected amniotic fluids. (A) PCA and (B) OPLS-DA displaying separation between transmitters and nontransmitters groups. (C) Volcano plot analysis to identify differentially abundant metabolites in the transmitters versus nontransmitters comparison and (D) their hierarchical clustering with heatmap highlighting abundance patterns in the transmitters group. (E) PCA and (F) OPLS-DA displaying separation between nontransmitters and CTRL groups. (G) Volcano plot analysis to identify differentially abundant metabolites in the nontransmitters versus CTRL comparison and (H) their hierarchical clustering with heatmap highlight of abundance patterns in the nontransmitters group.

**Table 3:** Differentially Abundant AF Metabolites in the Transmitters *versus* Nontransmitters Comparison^a^

Metabolite	Mean of transmitters	Mean of nontransmitters	Fold change	SE of difference	–log10 (*q*-value)
C4 (Butyrylcarnitine)	–3.619	–3.036	–0.5824	0.1105	3.665
Xle (Leucine+Isoleucine)	3.963	4.442	–0.4796	0.06857	5.381
Orn (Ornithine)	1.77	2.178	–0.4079	0.05103	5.745

a Metabolites were ordered by crescent Fold Change values.

Hence, to confirm such a hypothesis, we compared the AF metabolome of nontransmitters samples with that of healthy women (CTRL, *n* = 14). One CTRL sample was identified as an outlier and then excluded for further univariate and multivariate statistics. PCA and PERMANOVA analyses showed increased variance between the metabolic profiles analyzed (*F*-value = 65.045; *p*-value = 0.001) (Figure 1E). Accordingly, the OPLS-DA depicted a good separation between the different groups reporting a model with robust fitness (R2Y = 0.771) and high predictive ability (Q2 = 0.744) (Figure 1F). The global comparison of nontransmitters versus CTRL samples by univariate volcano plot analysis revealed a significant (*q* < 0.01) decrease of 17 analytes and an increase of one compound in the nontransmitters group (Figure 1G and Table 4). The 18 differentially abundant metabolites were reported in a heatmap showing the quantitative variations across the replicates and sample group clustering (Figure 1H).

**Table 4:** Differentially Abundant AF Metabolites in the Nontransmitters *versus* CTRL Comparison^a^

Metabolite	Mean of nontransmitters	Mean of CTRL	Fold Change	SE of difference	–log10 (*q*-value)
Glu (Glutamate)	4.573	6.375	–1.801	0.129	13.48
C6 (Hexanoylcarnitine)	–6.564	–5.685	–0.879	0.1083	8.659
C2 (Acetylcarnitine)	0.1815	1.04	–0.8581	0.05928	17.31
C18:2 (Octadecadienylcarnitine)	–7.411	–6.581	–0.83	0.198	3.515
Xle (Leucine+Isoleucine)	4.442	5.111	–0.6689	0.07928	9.276
Met (Methionine)	2.11	2.742	–0.632	0.06046	13.48
C0 (Carnitine)	2.061	2.669	–0.6079	0.04805	14.97
Arg (Arginine)	3.05	3.63	–0.58	0.08507	7.673
Tyr (Tyrosine)	3.733	4.301	–0.5673	0.07771	8.151
Phe (Phenylalanine)	3.593	4.143	–0.5505	0.05986	11.56
Orn (Ornithine)	2.178	2.722	–0.5443	0.07258	8.703
C5 (Valerylcarnitine)	–4.426	–3.937	–0.4893	0.09141	5.428
C16 (Palmitoylcarnitine)	–7.671	–7.193	–0.4781	0.1429	2.571
Ala (Alanine)	5.829	6.29	–0.4609	0.09023	4.646
Asp (Aspartate)	2.137	2.572	–0.435	0.06494	7.583
C8 (Octanoylcarnitine)	–6.258	–5.874	–0.3842	0.1128	2.559
Cit (Citrulline)	1.093	1.474	–0.381	0.08658	3.751
C5DC (Glutarylcarnitine)	–5.985	–6.513	0.5285	0.1365	3.207

a Metabolites were ordered by crescent Fold Change values.

Therefore, AF metabolomics showed the existence of significant differences between nontransmitters and controls, indicating early alterations of the AF metabolic profile even in the absence of fetal viral transmission.

To further strengthen our findings, we performed a three-group analysis including transmitters, nontransmitters, and CTRL samples. PCA analysis through PERMANOVA statistics (*F*-value = 46.432; *p*-value = 0.001) suggested high variance in the three metabolic profiles (Figure 2A). However, as illustrated in the PLS-DA model, the transmitters and nontransmitters groups are almost overlapping, being separated from the CTRL group (Figure 2B). These models suggest that the metabolic alterations may be early induced by CMV maternal infection regardless of transmission to the fetus.

**Figure 2:**
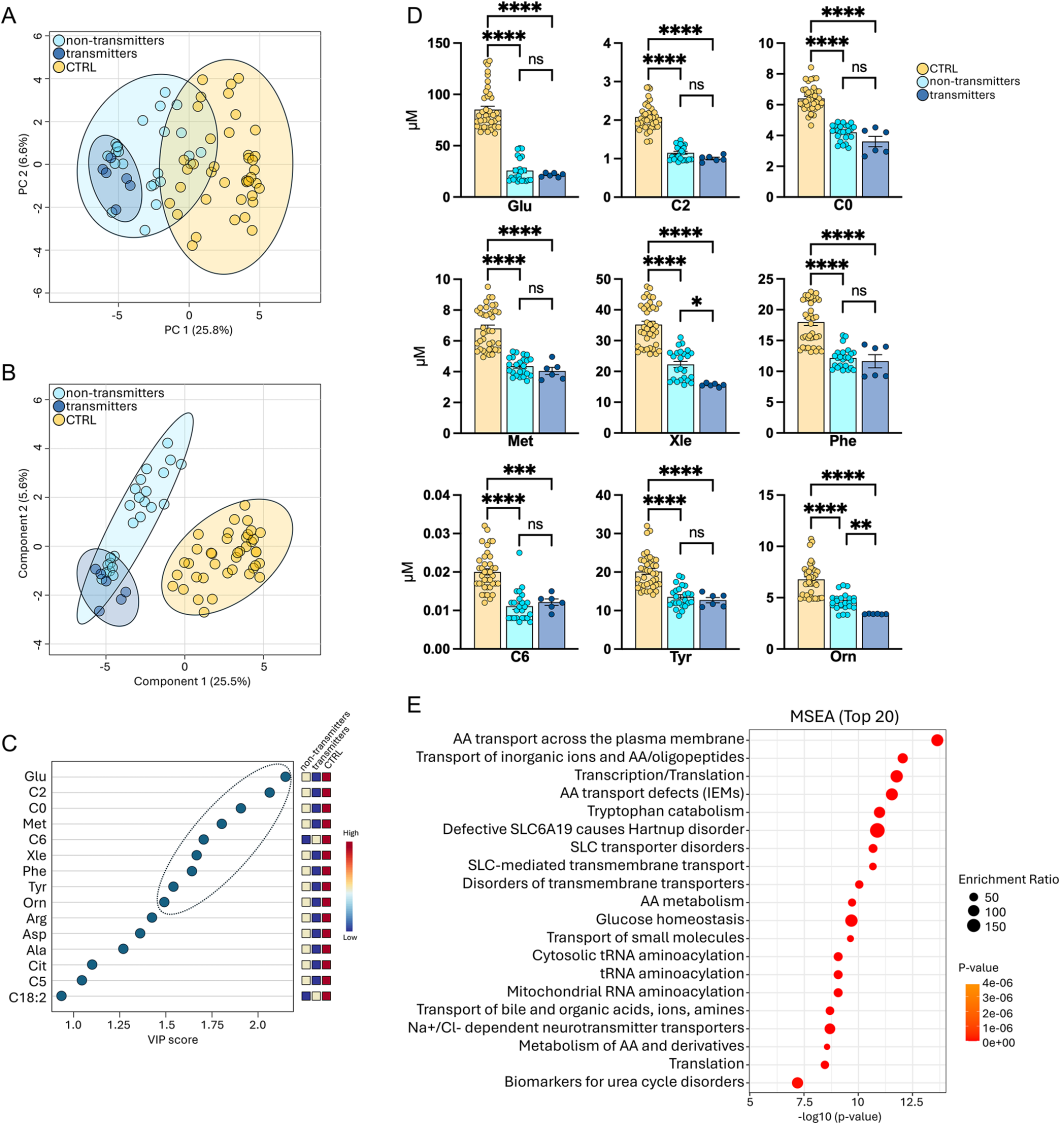
Metabolite statistical validation and pathway enrichment analysis. (A) PCA and (B) PLS-DA models to explain the separation between nontransmitters, transmitters, and CTRL groups. (C) VIP metabolites analysis based on the three-group PLS-DA model. Metabolites with VIP > 1.5 were considered important. (D) Univariate statistics for multiple comparisons to validate VIP metabolite dysregulations was carried out by ordinary one-way ANOVA; ****p < 0.0001, ***p < 0.001, **p < 0.01, *p < 0.05, ns = not significant. (E) MSEA performed through the over-representation analysis highlighted the top 20 pathways enriched by the VIP metabolites as input within the RaMP-DB (integrating KEGG via HMDB, Reactome, WikiPathways) pathway database.

For this reason, we extracted from the PLS-DA model a subset of VIP features that are useful to select the important variables that contribute the most to the metabolic phenotype (Figure 2C). In particular, Glu, C2 (VIP > 2), C0, Met, C6, Xle, Phe, Tyr, and Orn (VIP > 1.5) were selected as important molecules. All the VIP metabolites were among the differentially abundant species in the nontransmitters versus CTRL comparison (Figure 1G and Table 4), and the quantitative variation of the VIP molecules was validated by ANOVA statistics using the nontransformed concentration data (μM) obtained by the targeted MS analysis. All these molecules were confirmed to be increased in CTRL and decreased in both nontransmitters and transmitters samples (Figure 2D). Only Xle and Orn levels were different between nontransmitters and transmitters, as already confirmed by volcano plot analysis (Figure 1C).

Subsequently, altered biochemical pathways associated with the VIP signature were identified by metabolite set enrichment analysis (MSEA), showing interesting biological terms connected to VIP metabolites and associated with their dysregulation including amino acid transport, amino acid defects, glucose homeostasis, and metabolic disorders (Figure 2E).

### Biomarker Analysis Revealed the Strong Association of Glutamate and Acetylcarnitine

With the assumption that CMV may induce persistent changes regardless of its transmission to the fetus or its resolution, our study focused on identifying metabolic patterns that were consistently altered in both CMV-transmitters and nontransmitters. Given the overlap of transmitters and nontransmitters metabolomes, a multivariate ROC curve-based exploratory analysis was performed on the total CMV data set (transmitters + nontransmitters) compared to CTRL samples. To test the capability of one or multiple metabolites to correctly classify the samples to their group, ROC analysis was performed using the PLS-DA method for classification and feature ranking. Precisely, the combination of two metabolites in the exploratory ROC curves indicates the maximum confidence of differentiation in the CMV compared to the CTRL group, representing the best model with the AUC = 0.927 (Figure 3A). The significant features identified in the biomarker analysis are reported in Figure 3B, with highlights of the two features that best describe the classification model, namely, Glu and C2. Representative curves of Glu (AUC = 1) and C2 (AUC = 0.999) are shown in Figure 3C,D, and both metabolites were downregulated in CMV (transmitters + nontransmitters) compared to CTRL AF.

**Figure 3:**
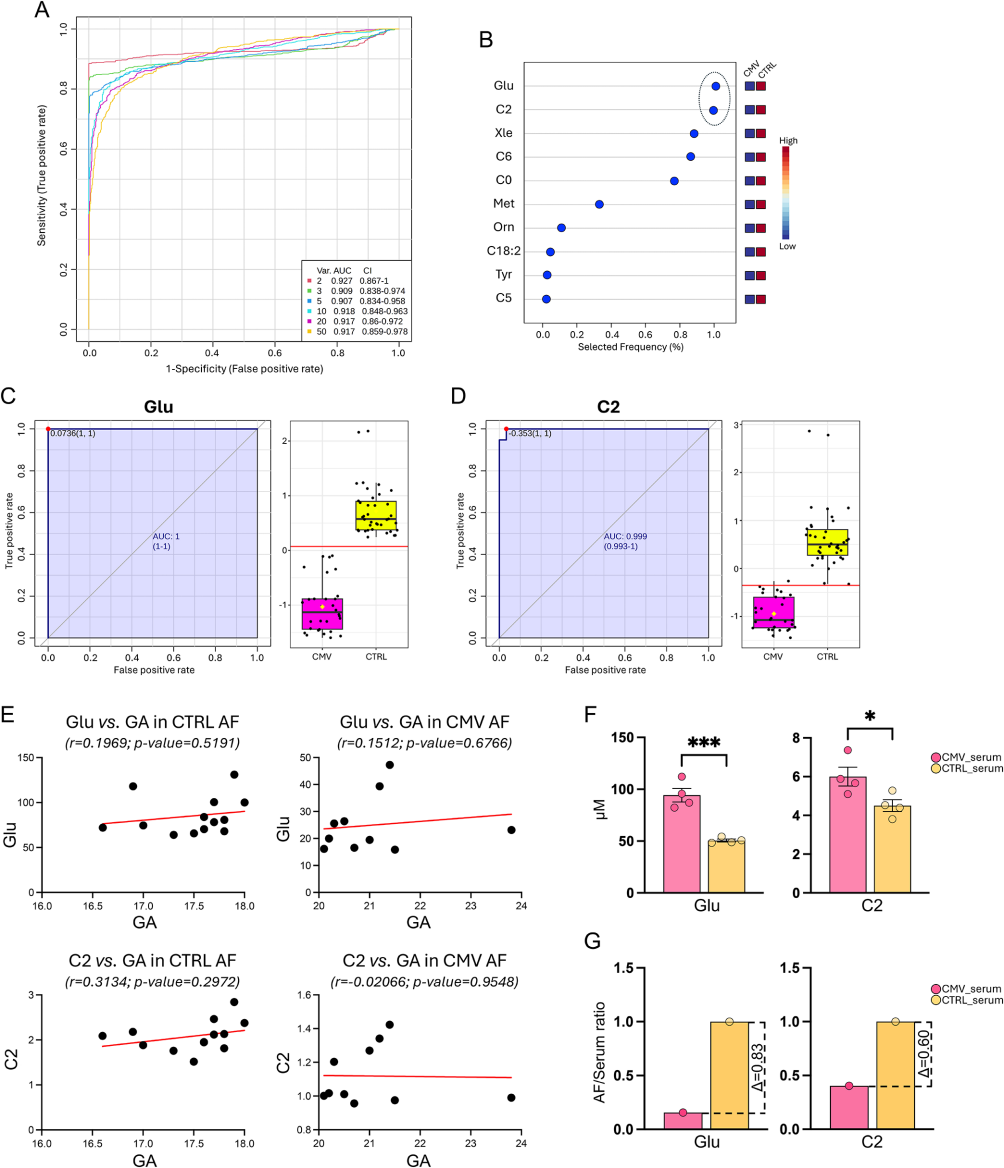
Biomarker evaluation in AF between CMV-exposed and CTRL samples. (A) ROC curve was generated by the PLS-DA model, with AUC values calculated from the combination of 2, 3, 5, 10, 20, and 50 metabolites. (B) Frequency plot showing 10 positively identified significant altered metabolites between CMV and CTRL groups. Representative AUC for two significantly downregulated metabolites in both the CMV subgroups, namely (C) glutamate (Glu) with AUC = 1 and (D) acetylcarnitine (C2) with AUC = 0.999. (E) Statistical Pearson correlation of Glu and C2 levels was performed in both the CMV and CTRL groups with their respective gestational age (GA) at the time of amniocentesis. (F) LC-MS/MS-based quantification of serum Glu and C2 levels from CMV-exposed and CTRL pregnant women. Statistical difference was assessed by unpaired *t*-test; ***p < 0.001, *p < 0.05. (G) AF/serum ratio was calculated as the ratio between the Glu or C2 levels in both the compartments. Δ values were the differences between CTRL and CMV ratios.

To confirm that the metabolic differences related to Glu and C2 were not due to the diverse amniocentesis timing of CTRL and CMV-exposed groups, we tested the statistical correlation of their levels in both CMV and CTRL samples with their respective GA at the time of amniocentesis. Both the metabolites showed absence of correlation with GA in CMV (Glu: *r* = 0.1512; *p*-value = 0.6766; C2: *r* = −0.02066; *p*-value = 0.9548) and CTRL (Glu: *r* = 0.1969; *p*-value = 0.5191; C2: *r* = 0.3134; *p*-value = 0.2972) groups (Figure 3E), thus excluding biases in metabolite comparison due to phenomena like fetal urine dilution.

Finally, we analyzed the serum of CMV-positive and CTRL pregnant women to measure the circulating Glu and C2 levels. LC-MS/MS analysis revealed that the CMV group presented higher serum levels of both the metabolites than CTRL (Figure 3F).

To assess whether these changes were associated with an altered distribution between the maternal and intrauterine compartments, we calculated the AF/serum ratios for Glu and C2 in both groups. The AF/serum ratio of both Glu and C2 levels was markedly reduced in the CMV group relative to controls, indicating a disproportionate decrease of these metabolites in AF despite their higher levels in maternal circulation (Figure 3G). The resulting Δ values clearly highlighted the diverging metabolic changes in the fetal and maternal compartments (Figure 3G).

## Discussion

High-dimensional omics approaches are commonly applied to shed light on the mechanisms underlying various diseases. Metabolomics is a powerful approach based on MS or NMR technologies to characterize molecules, providing quantitative and qualitative details of low-molecular-weight metabolites (≤1 kDa) in many biological matrices.^57,58^ It can disclose metabolic interactions even occurring within a semiclosed compartment such as the placenta.^59^ Therefore, profiling the AF metabolome of first-trimester parturients exposed to infection by viral agents such as HCMV may identify specific biochemicals and pathways responsible for early functional changes in the fetus with high sensitivity and precision.

AF is a highly dynamic and complex medium that plays a crucial role in fetal development by providing mechanical protection, nutrients, and a supportive environment for growth. In the early stages of gestation, its composition closely resembles maternal plasma, which diffuses through the fetal membranes. Between the 10th and 20th week, free and bidirectional diffusion between the fetus and the amniotic sac makes the fetal plasma similar to AF. Therefore, the analysis of AF composition before skin keratinization reflects the physiological state of the developing fetus.^60^

We obtained a characterization of the AF metabolome from pregnant women diagnosed with CMV infection in the first trimester of pregnancy and transmission to the fetus (transmitters), in comparison with uninfected fetuses (nontransmitters) and healthy controls. The low discriminative power between the metabolomic profiles of transmitters and nontransmitters can be attributed to the low fitness observed in the proposed model that showed high similarity between the two conditions, suggesting that further refinement or alternative analytical approaches may be required to improve classification accuracy. The same behavior between transmitters and nontransmitters was also observed in another AF metabolomics-based study.^61^ Conversely, metabolomic profiles of nontransmitters compared to CTRL revealed massive differences, suggesting that CMV-induced dysregulations may be reflected in AF even in fetuses with negative PCR results. Such differences are not due to the diverse amniocentesis timing of CTRL and CMV-exposed groups, thus excluding biases in metabolite comparison due to phenomena such as fetal urine dilution. We identified a specific signature of VIP metabolites, among which Glu and C2 were less abundant in both transmitters and nontransmitters than controls. These findings suggest a lower availability of these metabolites during the first trimester, aligning with the high energetic and biosynthetic demands imposed by HCMV. Studies have indicated that CMV reprograms host cell metabolism to institute its own specific metabolic program,^62,63^ enhancing glucose uptake and glycolytic flux.^64^ However, the virus may successfully replicate in glucose-free cultures through metabolic compensation in diverse metabolic niches.^65^ The host lipid metabolism can be reprogrammed as well to increase the flux from glucose to acetyl-CoA and malonyl-CoA, precursor for fatty acid synthesis.^66–69^ Despite enhanced glycolysis, redirecting glucose-derived carbons toward fatty acid synthesis requires compensatory anaplerotic substrates for the Krebs cycle function. In particular, glutamine (Gln) uptake and glutaminolysis (deamination to Glu by glutaminase) are boosted in infected cells, while glutamate dehydrogenase converts Glu into α-ketoglutarate, fueling the Krebs cycle.^70^ This metabolic rewiring is consistent with a modified Warburg effect, wherein glucose-derived carbons support biosynthesis over ATP production^70^ (Figure 4).

**Figure 4:**
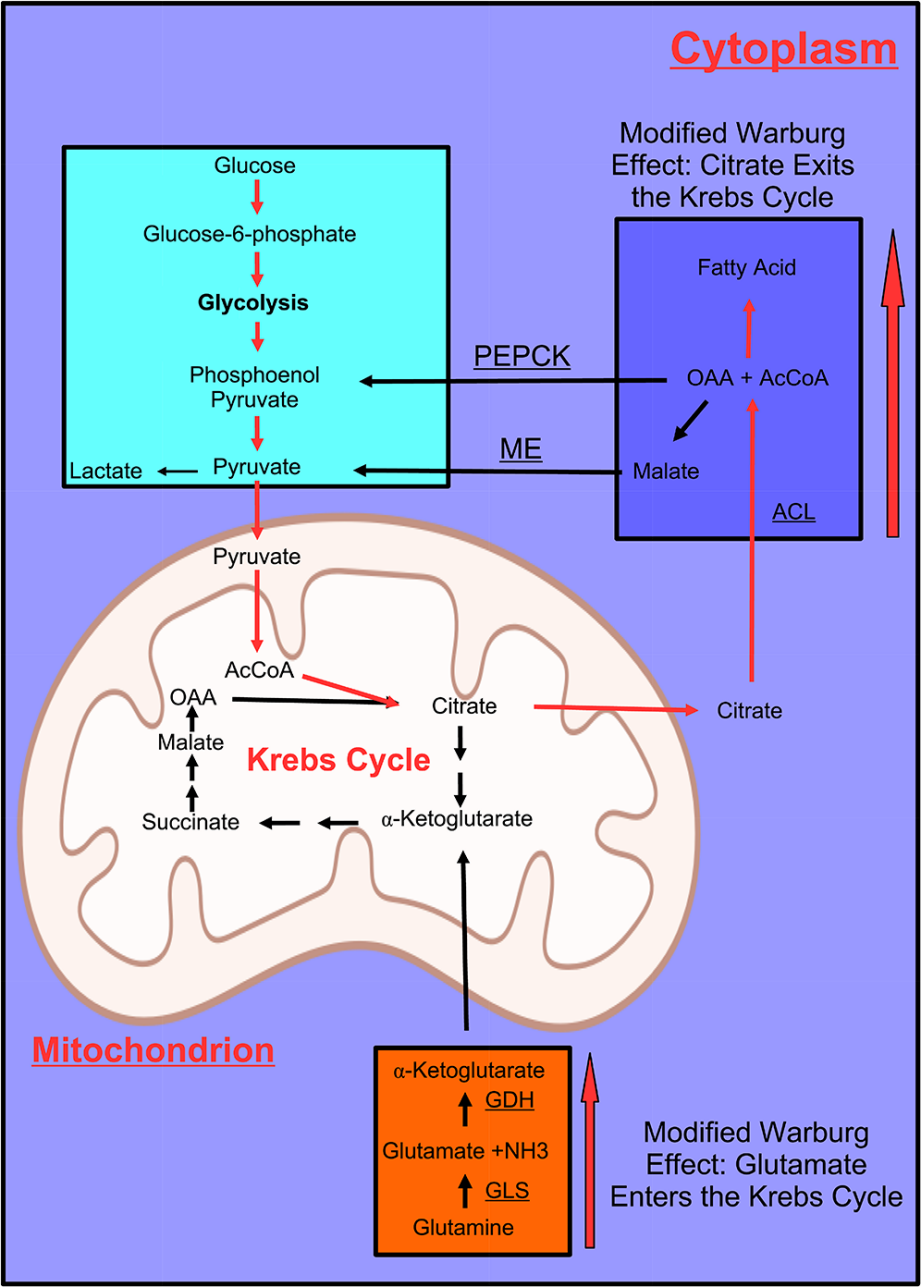
Modified Warburg effect: the figure schematizes the different utilizations of glucose and glutamate after CMV infection of cells, highlighting the aspects of cytoplasmic and mitochondrial metabolism discussed in the text. PEPCK, phosphoenolpyruvate carboxykinase; ME, malic enzyme; GDH, glutamate dehydrogenase; GLS, glutaminase; ACL, ATP citrate lyase; OAA, oxaloacetic acid; AcCoA, acetyl-coenzyme A. Figure drawn using the Biorender software.

In line with these concepts, our data may suggest that maternal CMV infection is associated with enhanced Gln utilization in AF and consequently in the fetal plasma, potentially reducing Glu availability for activities important to fetal glutaminergic neurons such as excitability, synaptic plasticity, immunity, learning, and memory.^37,71,72^ CMV-driven inflammatory and metabolic responses at the maternal-placental interface may modify Glu availability through alterations in uptake and transporter expression in human fetal astrocytes.^38^ Similar patterns of glutamatergic dysregulation have been reported in other neurotropic viral infections, including HIV-associated dementia^73,74^ and SARS-CoV-2-related astrocyte dysfunction^75,76^ both involving glutamatergic pathway disruption with metabolic rearrangement. Interestingly, our ROC analysis generated a model with high AUC that included Glu and C2 as surrogate biomarker signatures of recent CMV primary infection but not transplacental transmission. Our observation supports the hypothesis that metabolite depletion is an early and infection-driven event. The acetyl-L-carnitine C2 is an acetyl ester of carnitine with pleiotropic biological activities on the central and peripheral nervous system,^77^ and plays key roles in metabolism (glycogen production, β-oxidation, glucose utilization, ammonia cycling, etc.). C2 is actively transported into the brain, where it modulates aminergic neurotransmitter release and Glu biosynthesis/secretion, primarily via acetylation of the NF-κB p65 subunit.^78,79^ This enhances the transcription of the metabotropic glutamate receptor 2 (mGluR2), a G protein-coupled receptor that modulates synaptic plasticity, learning, memory, and emotional behaviors.^80^ mGluR2 acts as a Glu autoreceptor, attenuating presynaptic Glu release and neuronal excitability through negative feedback, thereby preventing excitotoxicity.^81^

Here, we highlighted diverging changes in Glu and C2 levels in the systemic circulation of CMV-exposed mothers and AF. Elevated Glu and C2 in the maternal serum during primary CMV infection may reflect enhanced systemic catabolism, immune activation, and mitochondrial stress.^82–84^ In contrast, their significant reduction in AF could reflect different mechanisms, including diminished Glu biosynthesis,^77,85–89^ impaired Glu release linked to mGluR2 signaling,^81^ or broader metabolic adaptations occurring at the maternal-fetal interface.^90,91^ As an example, because glutamatergic signaling regulates neuronal circuit refinement,^92^ Glu depletion may influence the synaptic pruning, which normally occurs during the last months of gestation to eliminate extra synapses and connections no longer needed^93^ (Figure 5). Other evidence shows that suboptimal Glu concentrations associated with enzymatic deficits of Glu metabolism can cause brain damage associated with various disorders, including gyrate atrophy, hyperammonemia, and organic acidurias.^94^

**Figure 5:**
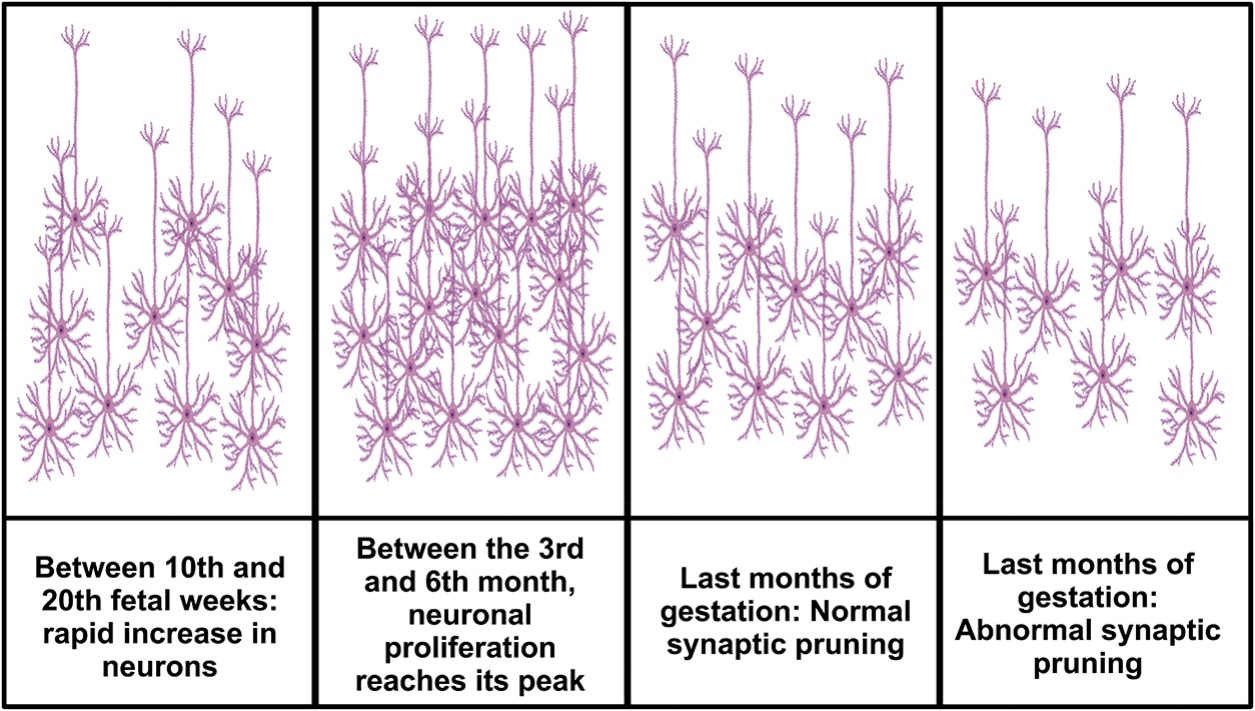
Schematic representation of the synaptic pruning evolution in the fetus, which may be different between CMV-infected and uninfected neural cells (healthy control). Figure drawn using the Biorender software.

Here, rather than indicating established neuronal injury, such metabolite depletion from the surrounding extracellular environment, which is still in open communication between the fetus and the amniotic compartment, may represent a shift in substrate utilization during a highly sensitive developmental period. Therefore, CMV-associated maternal metabolic stress might remodel the intrauterine biochemical environment before the full maturation of BBB, favoring the use of alternative fuels over glucose, with a metabolic shift resembling the modified Warburg effect (Figure 6).

**Figure 6:**
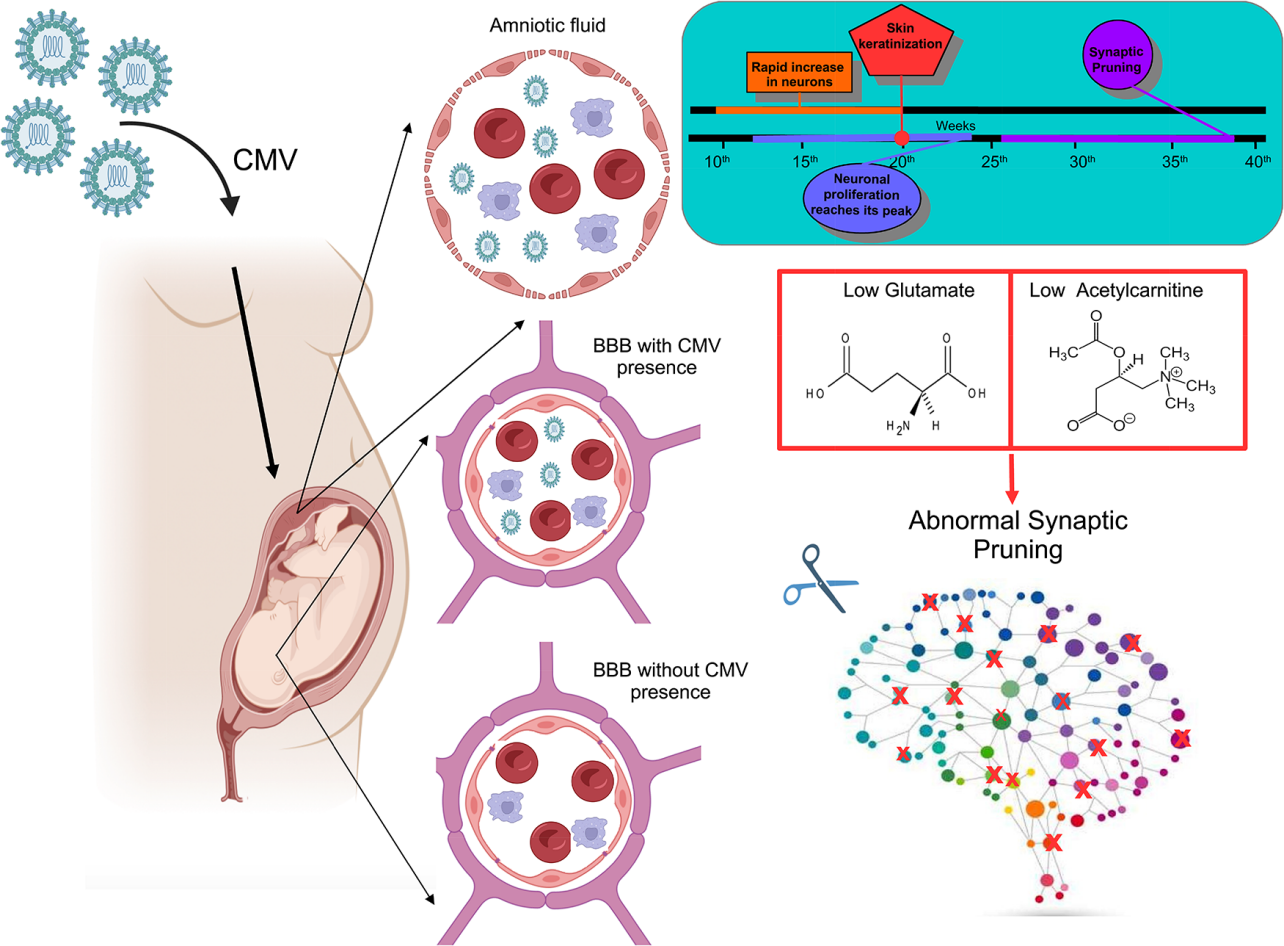
Representation of the hypothesis that depletion of glutamate and acetylcarnitine levels in the amniotic fluid of CMV-infected parturients in the first trimester of pregnancy may influence long-term processes in the fetus like the synaptic pruning, both in the case of CMV transmission or nontransmission through the blood–brain barrier. Figure drawn using the Biorender software.

We hypothesize that these metabolic changes reflect a broader metabolic reprogramming occurring at the maternal-placental interface where CMV activity and inflammation take place. Because AF is shaped by maternal metabolism, placental transport, and fetal metabolic-excretory activity, its profile may reflect metabolite synthesis/degradation, fetal maturation, and biochemical exchanges.^95^ The proposed mechanism relies on indirect metabolic consequences of maternal CMV infection, depending on the initial viral load and the GA at the time of infection, factors that also explain the high symptomatic variability present in infected newborns.^96^

At the same time, AF metabolomics may expand the current conceptual framework of HCMV infection during pregnancy, highlighting that maternal infections can influence the intrauterine biochemical environment beyond pathogen transmission. This perspective aligns with the concept of fetal programming, which is increasingly recognized in the context of prenatal infections and maternal inflammation, and according to which early consequences of maternal immune activation and placental-fetal metabolic adaptation are not fully captured through the viral load measurements alone.^40,41^ Our findings suggest that maternal CMV infection is associated with a distinct metabolic signature in the amniotic compartment, consistent with a state of altered maternal-fetal metabolic homeostasis. Nonetheless, several studies related to other infectious contexts highlight systemic maternal immune/metabolic responses to infection, including diverse effects on placental transport and fetal metabolic regulation that may lead to late-onset sequelae.^97–100^

In conclusion, the global changes of AF metabolites and the apparent dissociation between metabolic alterations and fetal infection status advocate metabolic programming arising from exposure to an altered intrauterine environment. We found out that transmitters and nontransmitters show an almost overlapping metabolome, of which Glu and C2 stand out as important downregulated molecules, suggesting that maternal infection alone is sufficient to induce metabolic remodeling at the maternal-fetal interface. We propose that such alterations may represent subclinical modifications of neurobiochemical pathways during sensitive developmental windows, the long-term significance of which remains unknown. This would open a new perspective to explain the known variability of congenital CMV outcomes as epidemiological evidence shows that some neurodevelopmental sequelae, such as late-onset hearing loss, can emerge months or years after birth despite initially asymptomatic presentations.

## Limitations of the Study

While this work offers valuable insights for clinical practice related to CMV infection during the first trimester of pregnancy, we acknowledge some limitations that may affect the generalizability of our findings, such as the size of our cohort. Amniocentesis is an invasive procedure typically reserved for specific diagnostic or therapeutic indications, which are determined by clinical suspect, genetic counseling, maternal age, and other risk factors. Besides the specific applicability, it provides increased risk of fetal injury, miscarriage, and fetal loss, making its use in current clinical practice relatively restricted. As a result, access to AF specimens is limited, constraining the size and scope of the research studies.

Another inherent limitation concerns the unavoidable time lag in AF sampling between CMV-infected and control pregnancies. In healthy pregnancies, amniocentesis is usually performed, when indicated, between the 15th and 20th weeks of gestation, when the uterus is adequately developed and sufficient AF volume is available. In contrast, for women with confirmed CMV infection, AF collection must be postponed at least 8 weeks after seroconversion, and typically not before the 20th week of pregnancy, to ensure reliable viral detection. Ethical and deontological constraints preclude the possibility of either delaying the procedure in controls or anticipating it in infected cases, resulting in an unavoidable temporal gap between experimental groups.

Moreover, our analysis was based on a targeted mass spectrometry approach, which inherently limits the range of detectable metabolites. In fact, untargeted metabolomics analyses may allow for a more comprehensive assessment of CMV-induced metabolic alterations. Given the widespread prevalence of CMV infections, future studies in larger independent cohorts are warranted to validate our findings and to further explore the metabolic disruptions associated with CMV using more expansive metabolomics platforms.

## Data Availability

Metabolomics data have been deposited at MetaboLights with the identification code MTBLS12415 and are publicly available as of the date of publication.
